# The Macrophage Galactose-Type Lectin-1 (MGL1) Recognizes *Taenia crassiceps* Antigens, Triggers Intracellular Signaling, and Is Critical for Resistance to This Infection

**DOI:** 10.1155/2015/615865

**Published:** 2015-01-15

**Authors:** Daniel Montero-Barrera, Héctor Valderrama-Carvajal, César A. Terrazas, Saúl Rojas-Hernández, Yadira Ledesma-Soto, Laura Vera-Arias, Maricela Carrasco-Yépez, Lorena Gómez-García, Diana Martínez-Saucedo, Mireya Becerra-Díaz, Luis I. Terrazas

**Affiliations:** ^1^Unidad de Biomedicina, Facultad de Estudios Superiores-Iztacala, Universidad Nacional Autónoma de México (UNAM), Avenue de los Barrios 1, Los Reyes Iztacala, 54090 Tlalnepantla, MEX, Mexico; ^2^Department of Pathology, The Ohio State University Medical Center, Columbus, OH, USA; ^3^Sección de Estudios de Postgrado e Investigación, Escuela Superior de Medicina, Instituto Politécnico Nacional, Mexico; ^4^Proyecto CyMA, UIICSE, FES Iztacala, UNAM, 54090 Tlalnepantla, MEX, Mexico; ^5^Instituto Nacional de Cardiología “Ignacio Chávez,” 14080 Mexico, DF, Mexico

## Abstract

C-type lectins are multifunctional sugar-binding molecules expressed on dendritic cells (DCs) and macrophages that internalize antigens for processing and presentation. Macrophage galactose-type lectin 1 (MGL1) recognizes glycoconjugates expressing Lewis X structures which contain galactose residues, and it is selectively expressed on immature DCs and macrophages. Helminth parasites contain large amounts of glycosylated components, which play a role in the immune regulation induced by such infections. Macrophages from MGL1^−/−^ mice showed less binding ability toward parasite antigens than their wild-type (WT) counterparts. Exposure of WT macrophages to *T. crassiceps* antigens triggered tyrosine phosphorylation signaling activity, which was diminished in MGL1^−/−^ macrophages. Following *T. crassiceps* infection, MGL1^−/−^ mice failed to produce significant levels of inflammatory cytokines early in the infection compared to WT mice. In contrast, MGL1^−/−^ mice developed a Th2-dominant immune response that was associated with significantly higher parasite loads, whereas WT mice were resistant. Flow cytometry and RT-PCR analyses showed overexpression of the mannose receptors, IL-4R*α*, PDL2, arginase-1, Ym1, and RELM-*α* on MGL1^−/−^ macrophages. These studies indicate that MGL1 is involved in *T. crassiceps* recognition and subsequent innate immune activation and resistance.

## 1. Introduction

Pattern recognition receptors (PRRs) function to rapidly detect pathogen invasion as well as to control innate immune activation leading to inflammation. The PRRs of dendritic cells (DCs) and macrophages (M*ϕ*s), among others, recognize and bind to conserved pathogen motifs such as LPS in gram-negative bacteria, flagellin, viral RNA, and several molecules in intracellular protozoa [1], including cyclophilin from* Toxoplasma gondii* [2] and GIPL from* Trypanosoma cruzi* [3]. Among the PRRs, the Toll-like receptor (TLR) family is the main group of receptors known to be involved in maturation and inducing inflammatory cytokines in DCs and M*ϕ*s [1]. Other types of PRRs are the C-type lectin receptors (CLRs), which have selective affinity for a carbohydrate or group of carbohydrates in a Ca^2+^-dependent manner; CLRs also play a role in recognizing different pathogens, including bacteria, fungal, and viral glycoconjugates [4–7]. However, there is a less-defined function for CLRs in response to helminth infections.

It has been largely demonstrated that helminths display a variety of glycan moieties on their antigens, which may participate in the induction of Th2 responses [8, 9] and in immune downregulation, both characteristics of helminth infections [10]. Nevertheless, given the complexity of these molecules, it may also be expected that some helminth glycoconjugates display immunostimulatory activities [11].

Some helminth parasites have been reported to be recognized by different CLRs, for example, dendritic cell-specific ICAM-3 grabbing integrin (DC-SIGN), mannose receptor (MR), macrophage galactose-type lectin (MGL), and SIGN-related 1 (SIGNR1) bind different glycans on soluble egg antigens from* Schistosoma mansoni in vitro* [12, 13].* Trichuris muris*, a nematode, is also recognized by the MR, but such CLRs appear to play a limited role in protection or susceptibility in both parasitic infections given that mice lacking the MR or SIGNR1 mount a normal immune response and clear these infections as usual [14].

*Taenia crassiceps* is a cestode that is useful for understanding the host-parasite relationship in cysticercosis. Glycoconjugates of* T. crassiceps* and other cestodes have been shown to induce strong Th2-biased responses* in vivo* [15] and to modulate both human and mouse DC activity* in vitro* [16–18]. We have previously reported that most of the* T. crassiceps* antigens bind to concanavalin A, indicating that they are glycosylated with glucose, mannose, or galactose [15, 17, 19]. The natural candidates for such carbohydrate recognition are CLRs, such as the MR, MGL, and DC-SIGN. As mentioned before, DC-SIGN and the MR have been shown to be irrelevant for* Schistosoma* or* Trichuris* protection. We therefore focused our study on MGL1. MGL1 is mainly found on M*ϕ*s and immature DCs, although it was recently reported to be expressed on mast cells [4], and it recognizes glycoconjugates expressing galactose and N-acetyl-galactosamine residues. Because the ability of MGL1 to act as an autonomous receptor has been appreciated only recently, the molecular regulation of its signaling pathway is partly known [20], but its biological role in response to helminth infections is still unrevealed. Recently, it has been shown that upon strong MGL engagement with polyclonal anti-MGL antibodies, this receptor is able to signal through tyrosine phosphorylation and induce ERK signaling [20], however, whether MGL signals after a “natural” ligation is also unknown.

To clarify the role of MGL1 in the recognition and defense against a helminth infection, we infected wild-type and MGL1-deficient mice (MGL1^−/−^) with* Taenia crassiceps*. Our data demonstrated that MGL1-deficient mice exhibited reduced parasite clearance, despite strong induction of Th2 responses. MGL1^−/−^ macrophages displayed reduced intracellular signaling in response to* Taenia* antigens and lower TNF-*α* and NO production. Taken together, our data suggest that MGL1 plays a key role in driving macrophage responses* in vivo* and thus may act as an important mediator of resistance to this helminth infection.

## 2. Materials and Methods

### 2.1. Mice

Six- to eight-week-old female MGL1^−/−^ mice on a C57BL/6 background were donated by Glycomics Consortium (USA). MGL1^−/−^ mice have been backcrossed for more than 7 generations on a C57BL/6NHsd genetic background with mice from Harlan Laboratories (México). In some experiments BALB/c mice were purchased from Harlan Laboratories (México). All mice were maintained in a pathogen-free environment at the FES-Iztacala, UNAM animal facilities, according to the Faculty Animal Care and Use Committee and government guidelines (official Mexican regulation NOM-062-ZOO-1999), which are in strict accordance with the recommendations in the Guide for the Care and Use of Laboratory Animals of the National Institutes of Health, USA.

### 2.2. Parasites and Infection

Metacestodes of* T. crassiceps* were harvested from the peritoneal cavity of female BALB/c mice after 2 to 3 months of infection. The cysticerci were washed four times in sterile phosphate-buffered saline (PBS) (0.15 M, pH 7.2). Experimental infection was achieved by intraperitoneal (i.p.) injection with 20 small (approx. 2 mm in diameter) nonbudding cysticerci of* T. crassiceps* suspended in 0.3 mL of PBS per mouse.

### 2.3. Lectin-Blot Analysis on* T. crassiceps* Soluble Antigens

*T. crassiceps* was lysed by sonication with one 10-s pulse at 100 W of amplitude (Fisher Sonic Dismembrator model 300). The resulting suspension was centrifuged at 4°C for 1 h at 2700 g, and the pellet was discarded and we keep the supernatant. Soluble antigen was concentrated and quantified by the Bradford method. A total of 20 *μ*g of protein of* T. crassiceps* soluble antigens (TcSol) was separated by SDS-PAGE (10%) and electroblotted (400 mA for 1 h) onto a nitrocellulose membrane [21].

For the detection of carbohydrates with residues of galactose or* N*-acetylgalactosamine, strips of nitrocellulose were blocked with 1% BSA diluted in PBS-T (10 mM sodium phosphate buffer, 150 mM NaCl and 0.5% Tween 20) and incubated overnight at 4°C. The membranes were treated with a biotinylated lectin (Hp or RCA) at a concentration of 5 *μ*g/mL diluted 1 : 500 in PBS-T and incubated overnight at 4°C (Sigma-Aldrich, St. Louis, MO, USA). The next day, the membranes were incubated with SBHP, the strips were washed with PBS, and enzyme activities were then detected by a substrate solution (0.1% H_2_O_2_, 17.5% methanol, 0.15% 4-chloro-*α*-naphthol and 82.5% PBS) following a 15-min incubation. The proteins that reacted with the lectins were identified on the nitrocellulose strips.

### 2.4. FITC Labeling of* T. crassiceps* Antigens

*T. crassiceps* soluble products (TcSol) were labeled with FITC (Sigma) according to the manufacturer's instructions. Briefly, 1 mg/mL TcSol was prepared in 0.1 M sodium bicarbonate buffer, pH 9. FITC was dissolved in DMSO at 1 mg/mL, and 500 *μ*L of the FITC solution was slowly added to TcSol in 5 *μ*L aliquots while gently stirring. The TcSol-FITC solution was incubated for 8 h at 4°C in the dark and washed 3 times with PBS; protein was quantified by the Bradford assay and maintained at 4°C.

### 2.5. Cell Preparations and Culture Conditions

The spleen was removed from infected mice under sterile conditions. Single-cell suspensions were prepared by gently teasing apart the spleen in RPMI-1640 supplemented with 10% fetal bovine serum, 100 units of penicillin/streptomycin, 2 mM glutamine, 25 mM HEPES buffer and 1% nonessential amino acids (all from GIBCO, BRL Grand Island, New York). The cells were centrifuged, and the erythrocytes were lysed by resuspending the cells in Boyle's solution (0.17 M Tris and 0.16 M ammonium chloride). Following two washes, the viable cells were counted by trypan blue exclusion with a Neubauer hemocytometer, and the splenocytes were adjusted to 3 × 10^6^ cells/mL in the same medium. Aliquots (100 *μ*L) of the adjusted cell suspensions were placed into 96-well flat bottom culture plates (Costar, Cambridge, Massachusetts) and stimulated with a soluble extract of* T. crassiceps* (25 *μ*g/mL) or with plate-bound anti-CD3 antibody (1 *μ*g/mL) at 37°C for 72 h. The supernatants from these cultures were analyzed for IL-4 and IFN-*γ* (PeproTech, México) production by ELISA.

### 2.6. Cytokine and Nitric Oxide Production by Peritoneal Macrophages

Peritoneal exudate cells (PECs) were obtained from uninfected thioglycolate-injected mice and from mice following 2, 4, and 8 weeks of* T. crassiceps* infection. PECs were adjusted to 5 × 10^6^/mL in supplemented RPMI and plated in 6-well plates (Costar). After 2 hours at 37°C and 5% CO_2_, nonadherent cells were removed and adherent cells were gently scrapped using cold PBS and readjusted to 1 × 10^6^/mL. Viability at this point was >90%. These cells were >90% macrophages according to FACS analysis (F4/80^+^, BioLegend, San Diego, CA). Then, 1 mL of the cell suspension was plated, and cells were activated in 24-well plates (Costar) with LPS (1 *μ*g/mL,* E. coli* 111:B4; Sigma, St Louis, MO.), followed by incubation for 24 h. TNF-*α*, IL-12 (PeproTech) and nitric oxide (Griess reaction) were examined in supernatants.

### 2.7. Generation of Bone Marrow Derived M*ϕ*s (BMDM*ϕ*s)

BMDM*ϕ*s from WT or MGL1^−/−^ mice were obtained as previously described [22]. Briefly, bone marrow cells were isolated by flushing femurs and tibias with culture media. Bone marrow cells were plated at 1 × 10^6^ cells/mL in medium supplemented RPMI medium supplemented with 10% SFB and penicillin/streptomycin plus 20 ng/mL murine recombinant macrophage colony-stimulating factor (M-CSF, Peprotech Mexico). On day 3, fresh media containing M-CSF was added. On day 7, nonadherent cells were removed, and adherent cells were detached and centrifuged at 1500 rpm at 4°C for 15 min. Adherent cells (F4/80^+^) were resuspended in fresh media and cultured for an additional 24 h in 12-well plates.

### 2.8. *In Vitro *Stimulation of BMDM*ϕ*s and Protein Extraction

Bone marrow cells were differentiated as described above. On day 7, the M*ϕ*s were harvested and plated at 2.5 × 10^6^ cells/mL in six-well plates. The cells were used 24 hours after plating to eliminate any residual effects from M-CSF. M*ϕ*s were stimulated with the TcSol (50 *μ*g/mL) for 5, 15, or 30 min. After stimulation, the cells were centrifuged at 1500 rpm for 5 min and washed with PBS. The cells were then lysed with lysis buffer for 15 min, and the lysates were quantified using the BCA assay (Thermo Scientific) and then frozen at −80°C until further use.

### 2.9. Western Blot Assays

Whole-cell lysates were resolved by SDS-PAGE (40 *μ*g of protein was loaded into each well) using 10% acrylamide mini-gels, followed by electrophoretic transfer to PVDF membranes (Immobilon-P MILLIPORE) for 2 h. Membranes were blocked with 5% fat-free milk in PBS for 2 h and incubated with primary antibodies overnight. The detection step was performed with peroxidase-coupled anti-mouse IgG and anti-rabbit IgG (BioLegend, 1 : 2000) and anti-goat IgG (Santa Cruz Biotechnology) for 2 h. The primary antibodies included antiphosphotyrosine (Santa Cruz Biotechnology) and *β*-Actin (BioLegend). All primary antibodies were diluted 1 : 500 in 1% fat-free milk in PBS. Blots were developed with the ECL detection system according to the manufacturer's instructions (Amersham). Blots are representative of two separate experiments.

### 2.10. Detection of Nitric Oxide Production

Nitric oxide production by macrophages was assayed by determining the increase in nitrite concentration [23] by the Griess reaction adapted to microwell plates (Costar). Briefly, 50 *μ*L of culture supernatant was mixed with an equal volume of Griess reagent and incubated for 10 min at room temperature in the dark; the absorbance was measured at 570 nm in an automatic microplate reader (Organon Technika Microwell System). Values were quantified using serial dilutions of sodium nitrite.

### 2.11. Flow Cytometry Analysis

Peritoneal exudate cells (PECs) were obtained from naïve or infected mice by injecting 10 mL of ice-cold PBS. For flow cytometry, single cell suspensions of the PECs obtained during the sacrifice were stained with anti-F4/80, anti-PDL2, anti-IL-4R*α*, anti-MGL1, and anti-MGL2 antibodies (Biolegend, San Diego, CA), for 30 min at 4°C. The cells were washed twice and analyzed using the FACSCalibur system and Cell Quest software (Becton Dickinson, USA).

### 2.12. Reverse Transcription (RT)-PCR Analyses

The levels of arginase 1 (Arg-1), Ym-1 and resistin-like molecule-*α* (RELM-*α*) mRNA transcripts in adherent peritoneal macrophages (which were allowed to adhere for 2 h at 37°C and 5% CO_2_) were determined using RT-PCR. At the indicated time points, adherent peritoneal macrophages from* T. crassiceps*-infected MGL1^+/+^ and MGL1^−/−^ mice were aseptically removed and without any further stimulation (basal) or LPS + IFN-*γ*-stimulation (1 *μ*g and 20 ng/mL, resp.) were processed for RNA extraction using TRIzol reagent (Invitrogen, Carlsbad, CA, USA) and the propanol-chloroform technique. The RNA was quantified, and 3 *μ*g of RNA was reverse transcribed using the Superscript II First Strand Synthesis Kit (Invitrogen) and an oligo dT primer, as recommended by the manufacturer. Once cDNA was obtained, conventional PCR was performed. The PCR reactions contained (in a 25 *μ*L final volume) 5X PCR Buffer blue, 10 mM dNTP, 40 nM each of the forward and reverse primers (Table 1), 1 U Taq DNA polymerase (Sacace Biotechnologies, Italy) and 2 *μ*L of the cDNA. The program used for the amplification of each gene contained an initial denaturation step at 95°C for 5 min; 35 cycles of 95°C for 40 s, the indicated melting temperature for 50 s and 72°C for 40 s; and a final extension step at 72°C for 4 min. All reactions were performed in a thermal cycler (Corbett Research, Australia). Finally, to observe the amplified products, a 1.5% agarose gel was prepared, and samples were loaded with blue juice buffer containing SYBR Green (Invitrogen). The gels were visualized using a Fujifilm FLA 5000 scanner (Fuji, Japan) with FLA 5000 image reader V2.1 software to capture the images. The specificity of the PCR was verified by the absence of signal in the no-template controls of macrophage samples. The sequences of the primers used have been previously reported [24].

### 2.13. Antibody ELISAs

Peripheral blood was collected at 2-wk intervals from tails snips of the* T. crassiceps*-infected MGL1^+/+^ and MGL1^−/−^ mice.* T. crassiceps*-specific IgG1 and IgG2a levels were determined by ELISA as previously described. The results are expressed as the maximal serum dilution (endpoint titer) at which Optical Density was detected (OD). Total IgE production was detected by Opt-ELISA from Biolegend.

### 2.14. Statistical Analysis

Comparisons between the wild-type and MGL1^−/−^ groups in this study were performed using Student's unpaired *t* test. *P* < 0.05 was considered significant. The statistical significance of the serum titers was determined by nonparametric tests using the Mann-Whitney *U*/Wilcoxon Rank tests.

## 3. Results

### 3.1. *Taenia crassiceps* Soluble Antigens Express Glycoconjugates Containing N-Acetylgalactosamine and Galactose Residues

**Figure 1:**
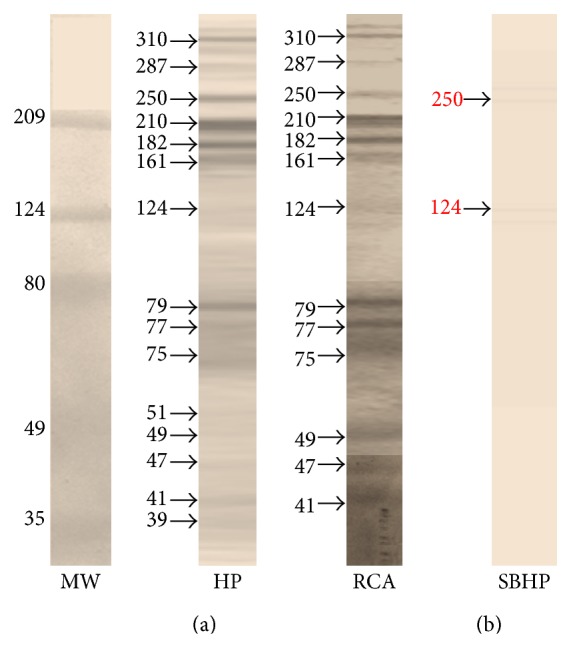
Lectin-blot analysis of* Taenia crassiceps* soluble antigens.* T. crassiceps* soluble proteins were subjected to electrophoresis on a 10% SDS-polyacrylamide gel, transferred to nitrocellulose membrane, and revealed with a couple of different biotinylated lectins. (a) MW-molecular weight, HP-*Helix pomatia* and RCA-*Ricinus communis*. (b) The strips were incubated only with SBHP-streptavidin-bound horseradish peroxidase.

To detect the expression of glycoconjugates on soluble antigens of* T. crassiceps*, a lectin-blot assay was performed using* Helix pomatia* (specific for N-acetylgalactosamine residues) and* Ricinus comunis* (specific for galactose residues) lectins. Several glycoconjugates were recognized by both lectins, those of 310, 287, 250, 210, 182, 161, 79, 77, 75, 49, 47, and 41 kDa (Figure 1(a)), whereas two bands at 51 and 39 KDa were only recognized by* H. pomatia*. However, using only streptavidin peroxidase (Figure 1(b)), two glycoconjugates were recognized on TcSol antigens, those of 250 and 124 kDa. Therefore, the presence of these two biotin-containing proteins most likely corresponds to biotin-dependent carboxylases recognized by streptavidin alone [25, 26]. These findings indicate that TcSol antigens express high levels of glycoconjugates with N-acetylgalactosamine and galactose residues.

### 3.2. MGL1^−/−^ Macrophages Display Reduced Binding to* Taenia crassiceps* Soluble Antigens

**Figure 2:**
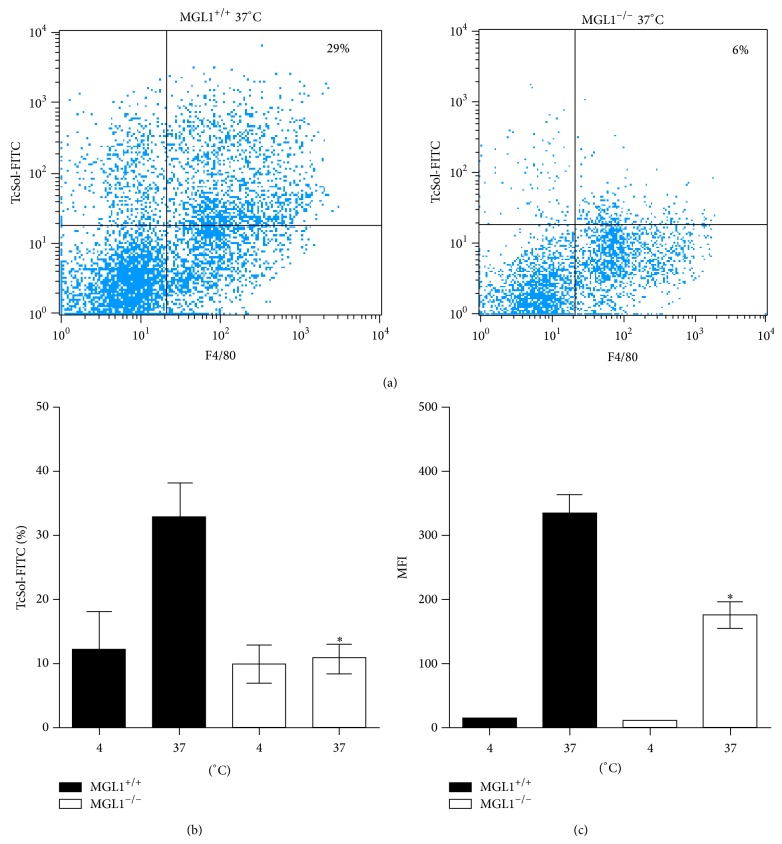
Peritoneal M*ϕ*s recognize* Taenia crassiceps* soluble products through MGL1. M*ϕ*s from naïve MGL1^+/+^ or MGL1^−/−^ mice were incubated with fluorescently labeled TcSol and fluorescence was analyzed by flow cytometry. (a) Representative dot blots indicate M*ϕ*s from either MGL1^+/+^ or MGL1^−/−^ mice, incubated for 30 minutes with TcSol at 37°C. (b) Percentage of TcSol-FITC positive naïve peritoneal cells exposed for 30 min. (c) Data are represented as the mean florescence intensity (MFI) of naïve M*ϕ*s exposed to TcES-FITC. Basal levels of autofluorescence were subtracted from all treatments. Data are representative of three independent experiments. ^*^ *P* < 0.05.

The innate recognition of pathogen-associated molecules by antigen-presenting cells is crucial to the initiation of the immune response. Different studies have shown that helminth-derived molecules can be recognized by C-type lectins [27, 28]. In our model, we previously reported that most TcSol bound to concanavalin A, indicating that TcSol are glycosylated with glucose, mannose or galactose [17], whereas Jang Lee et al. reported that the main N-glycan structures in* T. crassiceps* were mannose, fucose, galactose and GlcNAc. They also found a rare Fuc*α*1 → 3GlcNAc antenna on* T. crassiceps* molecules [29]. Thus, the natural candidates for carbohydrate recognition on* T. crassiceps* are CLRs, such as the MR, MGL, and DC-SIGN. Thus, we hypothesized that one of the possible receptors that bind the glycomolecules on TcSol may be MGL, given its specificity to recognize both N-acetylgalactosamine and galactose residues. To investigate the role of MGL1 in TcSol recognition, TcSol antigens were labeled with FITC to perform binding assays. TcSol-FITC efficiently bound to MGL1^+/+^ macrophages at 37°C (approximately 30% of cells were positive), indicating that TcSol-FITC can be recognized and internalized by these cells (Figures 2(a) and 2(b)). Incubation of M*ϕ*s and TcSol at 4°C showed reduced fluorescence (5–10%) indicating a low level of binding. Next, we similarly exposed MGL1^−/−^ M*ϕ*s to TcSol-FITC at 37°C and found significant reductions in both the percentage of TcSol-FITC-positive cells (approximately 10%) and the mean fluorescence intensity (MFI) of TcSol-FITC on these cells (Figure 2(c)). These data suggest that M*ϕ*s can recognize TcSol via MGL.

### 3.3. The Absence of MGL1 on Macrophages Impairs TcSol-Triggered Global Tyrosine Phosphorylation

**Figure 3:**
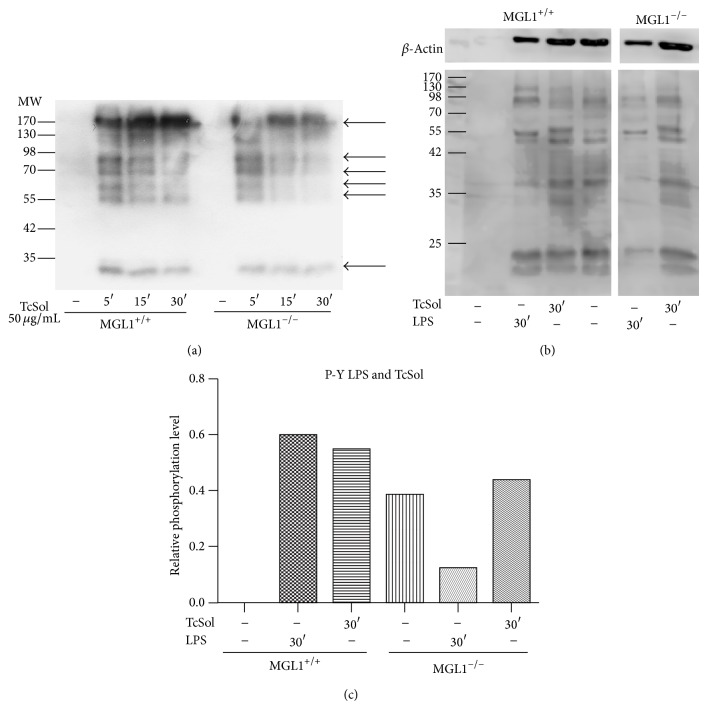
MGL1^−/−^ macrophages display deficient tyrosine phosphorylation in response to TcSol. (a) MGL1^+/+^ and MGL1^−/−^ bone marrow-derived M*ϕ* were synchronized in starvation conditions over night and then were stimulated or not with TcSol (50 *μ*g/mL) for 5, 15 or 30 min. Next, total cell extracts were obtained and resolved by electrophoresis, transferred to nitrocellulose, and probed with monoclonal antiphosphotyrosine antibody. Lane 1, serum-starved MGL1^+/+^ M*ϕ*s. Lanes 2–4, serum-starved MGL1^+/+^ M*ϕ*s following TcSol exposure for 5, 15 and 30 min, respectively. Lane 5, serum-starved MGL1^−/−^ M*ϕ*s. Lanes 6–8, serum-starved MGL1^−/−^ M*ϕ*s following TcSol exposure for 5, 15, and 30 min, respectively. Lanes 2–4 show an upregulation of the tyrosine phosphorylation of total proteins, which was sustained for at least 30 min (arrows). Lanes 6–8 show an unsustained tyrosine phosphorylation of proteins after similar TcSol stimulation of MGL1^−/−^ M*ϕ*s. (b) Similar experiment using LPS (500 ng/mL) and/or TcSol stimulation for 30 min. (c) Densitometry of western blot placed in (b). Proteins were visualized using a goat anti-mouse secondary conjugated to HRP and a chemiluminescence detection system. Western blots are representative of two independent experiments.

The fact that TcSol binding was impaired in MGL1^−/−^ M*ϕ*s, led us to question whether the interaction with MGL-TcSol triggers an intracellular signaling pathway. Recent advances in the understanding of the intracellular signaling induced by strong antibody-mediated crosslinking of MGL have highlighted a possible role for the ERK pathway [20]. To determine whether TcSol can induce intracellular signaling, we exposed BMDM*ϕ*s from MGL1^+/+^ or MGL1^−/−^ mice to TcSol for 5, 15, and 30 min and found that tyrosine phosphorylation was enhanced in MGL1^+/+^ macrophages; interestingly, tyrosine phosphorylation was detected as early as 5 min and remained for at least 30 min (Figure 3(a)). In contrast, MGL1^−/−^ BMDM*ϕ*s that were similarly exposed to TcSol displayed a lower and less-sustained tyrosine phosphorylation in several protein bands (Figure 3(a), arrows). To further analyze this response we stimulated both MGL1^+/+^ and MGL1^−/−^ BMDM*ϕ*s with LPS (500 ng/mL) or TcSol again for 30 min. As observed in Figure 3(b), also we detected a lack of response to LPS and a lower tyrosine phosphorylation in response to TcSol (Figures 3(b) and 3(c), resp.) in MGL1^−/−^ BMDM*ϕ*s.

### 3.4. Mice Lacking MGL1 Are Highly Susceptible to* Taenia crassiceps* Infection

After determining that* T. crassiceps* antigens can be recognized by MGL1 and that macrophages deficient in MGL1 are partially refractory to TcSol stimulation, we investigated the* in vivo* role of this molecule in experimental cysticercosis using MGL-deficient mice. To approach this question, we compared the course of* T. crassiceps* infection in MGL1^−/−^ with that in MGL1^+/+^ mice. As a control for susceptibility, we used BALB/c mice, which have consistently been reported as a highly susceptible strain to* T. crassiceps* infection [30].

**Figure 4:**
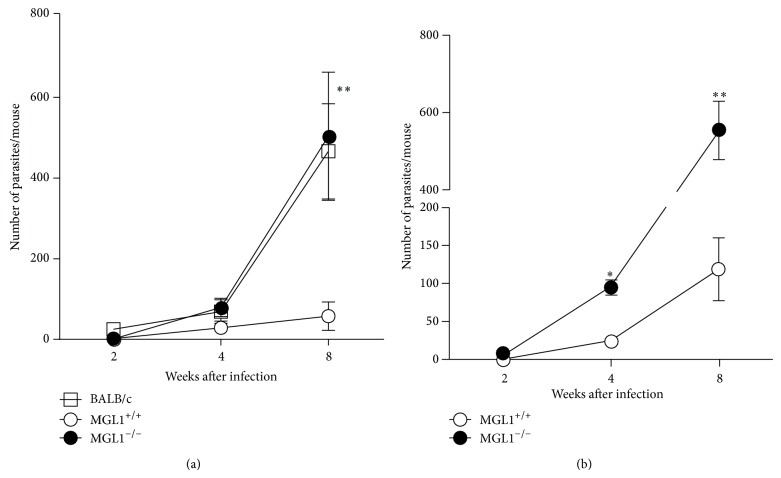
MGL1^−/−^ mice do not efficiently control* T. crassiceps *infection. (a) Course of i.p.* T. crassiceps *infection in MGL1^−/−^, MGL1^+/+^ and BALB/c mice after infection with 20 cysticerci. (b) An independent experiment of the course of i.p.* T. crassiceps *infection in MGL1^−/−^ and MGL1^+/+^ mice that were similarly infected. Data are expressed as the mean ± SE of 6 mice per group. ^*^ *P* < 0.01 comparing MGL1^−/−^ versus MGL1^+/+^ at the same time point.

We examined the kinetics of parasite growth from 2 to 8 weeks after* T. crassiceps* infection. Early in the infection (2 weeks) both groups of mice displayed comparable parasite burdens (Figure 4(a)). Interestingly, as infection progressed, by the fourth week of infection, the number of larvae in the peritoneal cavity increased significantly in MGL1^−/−^ mice compared to MGL1^+/+^ mice, which successfully reduced the number of parasites by week 8 after infection (Figures 4(a) and 4(b)). In fact, MGL1^−/−^ mice exhibited a parasite burden that was very similar to that in the susceptible strain of mice (BALB/c), which were consistently observed as very susceptible to* T. crassiceps* infection (Figure 4(a)). These findings suggest that the MGL1-mediated signaling pathway is involved in resistance during* T. crassiceps *infection on a resistant genetic background such as C57BL/6.

### 3.5. MGL1^−/−^ Mice Display an Altered Immune Response to* Taenia crassiceps*

A few studies have demonstrated that MGL can mediate signaling after a strong stimulus such as polyclonal anti-MGL antibodies, but its role in modulating protective immunity against helminth parasites is unknown. Therefore, we measured levels of Th1-associated IgG2a as well as Th2-associated IgG1 and total IgE antibodies in MGL1^−/−^ and MGL1^+/+^ mice at different time points following infection with* T. crassiceps*.

**Figure 5:**
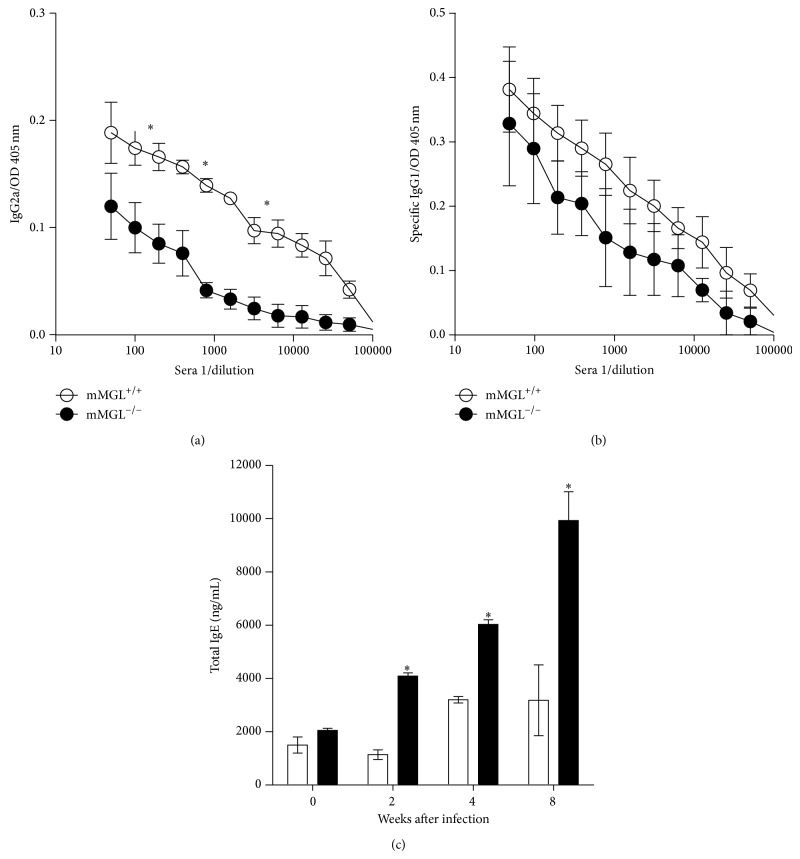
Antibody production during* T. crassiceps *infection in MGL1^−/−^ and MGL1^+/+^ mice. (a) Anti-*T. crassiceps*-specific IgG2a; (b) anti-*T. crassiceps*-specific IgG1; (c) total IgE. Values are the mean ± SE (*n* = 6 animals) and are representative of three independent experiments. ^*^ *P* < 0.05 comparing MGL1^−/−^ versus MGL1^+/+^ mice at the same time point.

Early in infection,* T. crassiceps*-infected MGL1^+/+^ and MGL1^−/−^ mice displayed comparable levels of* T. crassiceps* Ag-specific Th1-associated IgG2a antibodies (data not shown). However, by week 8 after infection, MGL1^+/+^ mice displayed significantly higher titers of specific IgG2a antibodies against* T. crassiceps* antigens (Figure 5(a)). By contrast, no clear differences were observed in Th2-associated IgG1 production; MGL1^+/+^ mice displayed high titers of anti-*T. crassiceps*-specific IgG1 that were similar to those in MGL1^−/−^ mice at week 8 after infection (Figure 5(b)). Although Th2-associated IgE has been shown to play a role in mediating immunity against certain helminths, we found that* T. crassiceps*-infected MGL1^+/+^ mice harbored a lower parasite burden despite producing significantly lower levels of IgE compared to similarly infected MGL1^−/−^ mice, which displayed higher levels of total IgE (Figure 5(c)).

**Figure 6:**
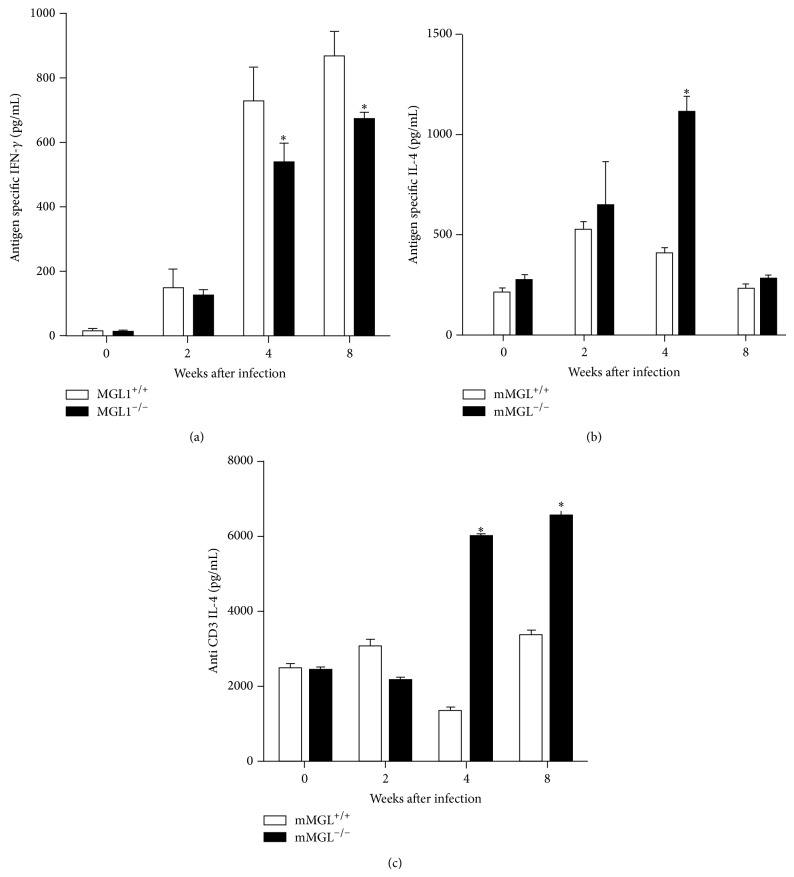
Kinetics of* in vitro* cytokine production by TcSol-stimulated spleen cells from MGL1^−/−^ and MGL1^+/+^ mice. (a) Antigen-specific IFN-*γ* production, (b) antigen*-*specific IL-4 production, and (c) polyclonal IL-4 production (anti-CD3) in response to* in vitro* stimulation with TcSol (25 *μ*g/mL) by splenocytes after 72 h. Data are representative of 2 independent experiments. ^*^ *P* < 0.05.

Additionally, we also compared the cytokine production by splenocytes from these mice in response to either 25 *μ*g/mL TcSol or 1 *μ*g/mL plate-bound anti-CD3 antibody. Anti-CD3 (data not shown) or TcSol-stimulated splenocytes from both strains produced similar levels of IFN-*γ* at 2 wk after infection (Figure 6(a)). However, 4 and 8 weeks after infection, the IFN-*γ* production by the spleen cells of MGL1^−/−^ mice decreased and did not reach the level produced by MGL1^+/+^ splenocytes (Figure 6(a)). Similarly, as early as 2 wk after infection, the splenocytes from the MGL1^−/−^ and MGL1^+/+^ mice produced comparable levels of IL-4 (Figure 6(b)). However, by weeks 4 and 8 after infection, MGL1^−/−^ mice produced significantly more IL-4 than the MGL1^+/+^ splenocytes in response to anti-CD3, whereas antigen-specific higher IL-4 production in MGL1^−/−^ splenocytes was only observed at week 4 after infection compared with MGL1^+/+^ cells (Figures 6(b) and 6(c)). Thus, as the infection progressed, splenocytes from MGL1^−/−^ mice produced significantly greater levels of IL-4 compared with splenocytes from* T. crassiceps*-infected MGL1^+/+^ mice in response to anti-CD3.

### 3.6. Differential Cytokine Production in MGL1^−/−^ and MGL1^+/+^ Peritoneal Macrophages

**Figure 7:**
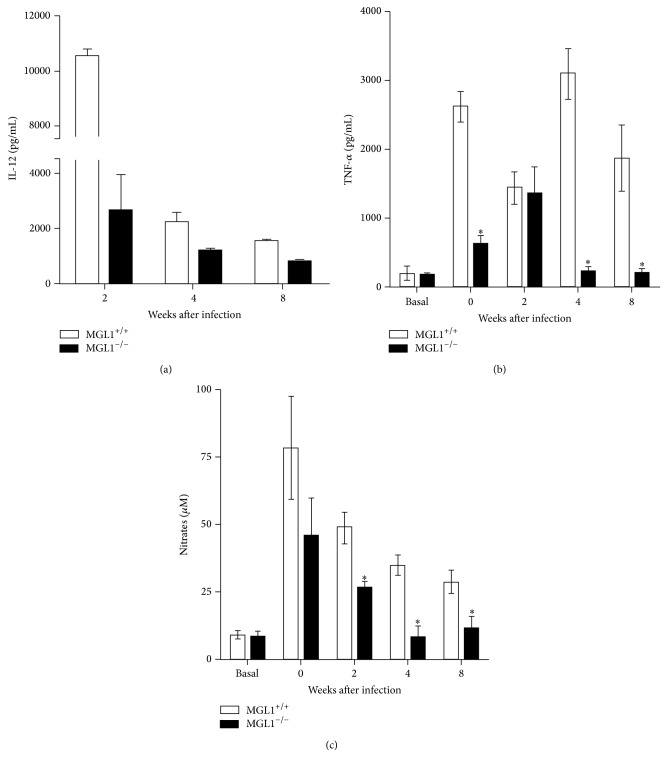
Peritoneal macrophages from MGL1^−/−^ and MGL1^+/+^ *T. crassiceps*-infected mice display different responses. Macrophages were obtained at different time points after infection and stimulated with LPS (1 *μ*g/mL) plus IFN-*γ* (5 ng/mL) for 48 h; supernatants were analyzed for (a) IL-12; (b) TNF-*α*; and (c) NO production. Data are expressed as in Figure 2. ^*^ *P* < 0.05. Data are representative of 2 independent experiments. ^*^ *P* < 0.05.

To determine whether MGL1^−/−^ mice had a systemic defect in innate activation, we examined the responsiveness of macrophages from MGL1^−/−^ mice to proinflammatory stimuli. Macrophages were isolated from the peritoneal cavities of both strains of infected mice and either left unstimulated (basal) or stimulated for 24 h with LPS (1 *μ*g/mL) and IFN-*γ* (20 ng/mL). The supernatants were collected and analyzed for IL-12, TNF-*α* and NO production. As shown in Figure 7, macrophages from MGL1^−/−^ mice obtained during the early phase of infection with* T. crassiceps* (2 wks) produced lower levels of IL-12 and TNF-*α* compared with those from MGL1^+/+^ mice (Figures 7(a) and 7(b)). As the infection became chronic, macrophages from infected MGL1^−/−^ mice produced decreased levels of TNF-*α* (Figure 7(b)), and the NO levels dropped significantly (Figure 7(c)) in both groups, but even more in MGL1^−/−^ macrophages. These patterns of macrophage response were in contrast to those observed in MGL1^+/+^ macrophages, which showed a better proinflammatory response throughout the infection with higher production of TNF-*α* and NO in late infections compared to MGL1^−/−^ macrophages (Figures 7(b) and 7(c)).

### 3.7. MGL1^−/−^ Mice Recruit Alternatively Activated Macrophages

**Figure 8:**
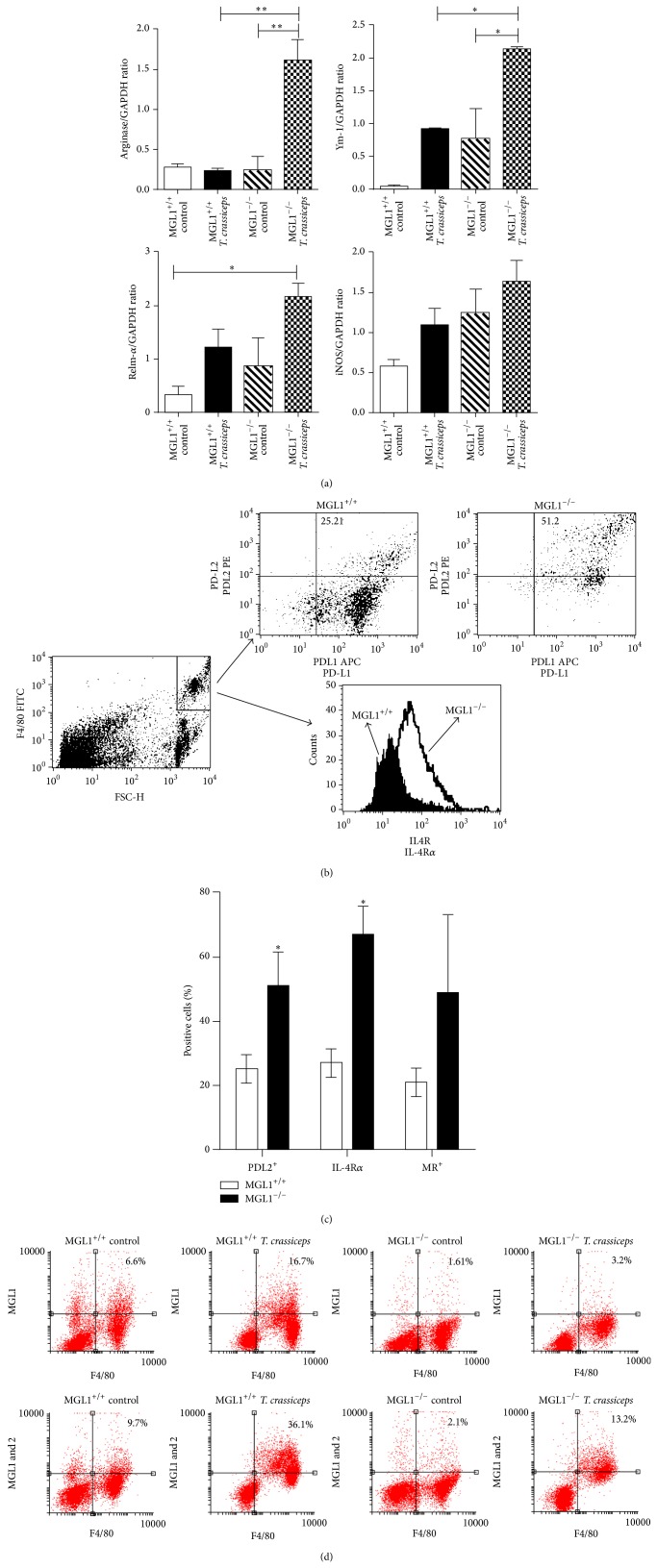
MGL1^−/−^ mice recruit alternatively activated macrophages. Peritoneal macrophages were obtained from MGL1^−/−^ and MGL1^+/+^ *T. crassiceps*-infected mice and processed for RT-PCR or flow cytometry analyses. (a) RT-PCR showing the expression of arginase-1, Ym1, RELM-*α* and iNOS on macrophages obtained at 8 weeks after infection with* T. crassiceps*. (b) Representative dot plot and histograms demonstrating increased expression of PD-L2, and IL-4R*α* on MGL1^−/−^ macrophages. (c) Percentages of expression of different surface markers on peritoneal macrophages. (d) Dot plots from PECs showing the expression of MGL1 and MGL2 from uninfected (control) and 8 weeks-infected* T. crassiceps* MGL1^+/+^ and MGL1^−/−^ mice, numbers on the quadrants indicate average percentage of positive cells. Values are the mean ± SE (*n* = 6 animals) and are representative of two independent experiments. ^*^ *P* < 0.05 comparing MGL1^−/−^ versus MGL1^+/+^ mice at the same time point.

Next, the macrophage polarization genotype was evaluated in adherent peritoneal cells at 4 and 8 weeks after infection. RT-PCR was performed to identify mRNA transcripts of AAM*ϕ*s markers. Infected MGL1^−/−^ mice showed higher expression of arginase-1, Relm-*α* and Ym-1 than their MGL1^+/+^ counterparts (Figure 8(a)). Moreover, we also analyzed the macrophage surface expression of other markers associated with alternative activation by flow cytometry. Surface expression of the MR, IL-4R*α*, and PD-L2 has previously been associated with AAM*ϕ*s in distinct helminth infections [31]. Indeed, we found differences between the two strains in the expression of these molecules during* T. crassiceps* infection. Interestingly, during chronic infection (8 weeks), the macrophages from MGL1^−/−^ mice expressed twice as much PD-L2 as macrophages from MGL1^+/+^ mice, which downregulated PD-L2 expression (Figures 8(b) and 8(c)). The MR and IL-4R*α* expression were also been significantly elevated in macrophages from* T. crassiceps*-infected MGL1^−/−^ mice when compared with MGL1^+/+^ mice that expressed lower percentages of both markers (Figures 8(b) and 8(c)).

Given that two related MGL molecules do exist in mice, MGL1 and MGL2, with different carbohydrate specificities, we performed flow cytometry on peritoneal cells for the detection of MGL1 and MGL2 in order to further determine the specific role for MGL1 and not for MGL2 in susceptibility to* T. crassiceps*. As shown in Figure 8(d), peritoneal macrophages (F4/80^+^) from MGL1^+/+^ infected mice did increase MGL1 as well as MGL2 expression after 8 weeks of infection, whereas MGL1^−/−^ macrophages were able to express MGL2 at a similar level than MGL1^+/+^ mice. These data suggest a very fine discrimination between MGL1 and MGL2 to recognize glycan structures on* T. crassiceps* and its antigens.

## 4. Discussion

The role of CLRs in the recognition of microbial, fungal, and parasitic glycoconjugates has become evident in the last few years [5]. The knowledge in this area has passed from understanding them as “simple endocytic receptors” to important molecules involved in innate immunity with the capacity to trigger intracellular signaling pathways to modify cellular responses to different pathogens [32]. Although the role for CLRs in immunity against fungal and bacterial infections is widely recognized, their role in immunity during helminth infections is much less known [13]. Therefore, in this study, we focused on the role of MGL1 and MGL1-mediated signaling in immunity to the cestode* T. crassiceps*.

It has been previously reported that* T. crassiceps* antigens are rich in structures recognized by concanavalin A. Here, we found that in addition to this and to the previously reported fucose [17, 29], glycosylation moieties in* T. crassiceps* such as galactose and N-acetylgalactosamine are also important. Because the carbohydrates in* T. crassiceps* antigens, as well as in those of other helminths, are considered important for their modulatory activities [17], we investigated the role of MGL in the ability to recognize glycosylated structures on TcSol as well as the possible intracellular signaling pathways that they may trigger. The lower recognition of TcSol by MGL1^−/−^ M*ϕ*s strongly suggested a role for this C-type lectin in the host-parasite interaction during experimental cysticercosis. Concomitantly, the decreased ability of MGL1^−/−^ macrophages to bind TcSol was associated with weak phosphotyrosine-mediated intracellular signaling. Therefore, we performed* in vivo* assays to determine whether MGL1 recognition of* T. crassiceps* plays a critical role in the resistance to this helminth infection. Thus, we demonstrate for the first time that mice deficient in the CLR macrophage galactose-type lectin 1 (MGL1) have increased susceptibility to experimental cysticercosis caused by the cestode* T. crassiceps*, with a reduction in the levels of the proinflammatory cytokines IL-12, IFN-*γ* and TNF-*α*, as well as in NO production. These observations are in line with those reported using anti-MGL antibody engagement on DCs, where MGL-engaged DCs produced higher levels of IL-10 than did DCs treated with the control isotype [20]. In contrast, higher levels of IL-4 and IgE were detected in these mice compared to similarly infected MGL1^+/+^ mice. Although Th2-associated responses and high levels of IgE correlate with protection in gastrointestinal helminth infections [33], our data suggest that IgE may have a limited role in mediating protective immunity against* T. crassiceps*. Notably, despite the greater Th2-associated antibody response in MGL1^−/−^ mice, these mice displayed greater susceptibility to* T. crassiceps*. These data agree with those reported by others [34, 35] and indicate a new and finely tuned role for MGL1 in activating innate and adaptive immune responses against this parasite. These results also suggest that the MGL1-dependent signaling pathway is not only required for inflammatory cytokine production in the early phase of the host response to* T. crassiceps* infection but also that MGL may play a critical role in the development of Th1-adaptive immunity, both of which may be essential for limiting infection and reducing pathology during experimental cysticercosis.

*In vivo*, MGL1^−/−^ mice significantly increased recruitment of alternatively activated macrophages, as determined by mRNA transcripts for arginase-1, Ym-1 and FIZZ1, and higher expression of the MR, IL-4R*α*, PD-L2 and MGL2 in their membranes. Thus, a clear alternative activation status in macrophages was observed only in MGL1^−/−^ mice. These findings are in line with previous reports showing alternatively activated macrophages associated with susceptibility in this model [24] and also indicate that MGL1 expression is not necessary for the induction of AAM*ϕ*s [36]. Together, these data indicate a role for MGL1 in the regulation of inflammation in a model of experimental cysticercosis and suggest that MGL1 may be a critical innate factor in the response to helminths and may be to other parasites.

Although many helminth parasites express a large number of carbohydrates, few CLRs have been associated with helminth recognition and* in vivo* function; for example, Dectin-2, DC-SIGN, SIGNR3, SIGNR1, and MGL have been shown to recognize several components of* S. mansoni* [13], but their roles* in vivo* are unclear. On the other hand, the MR has been associated with uptake of cercariae [12], but no one CLR has been demonstrated to be essential for resistance to such infection. Moreover, in another helminth infection, the absence of the MR during* Trichuris muris *challenge was irrelevant for either an immune response or resistance to this worm infection [14]. However, MR^−/−^ mice were recently demonstrated to exhibit increased survival to* Mesocestoides corti* infection, a neurocysticercosis model, mainly through the downregulation of inflammatory responses [37]. These observations highlight the role of different CLRs in addressing distinct helminth infections and also suggest that it is possible to identify differential roles of CLRs depending upon the type of helminth challenge.

The specificity of rodent MGL1 has been addressed in at least two papers [38, 39] where was shown MGL1 has a primary specificity for the Lewis X determinant, and it may additionally bind biantennary glycans ending in LacNAc or LacDiNAc. We have previously shown that Tcsol contains glycoconjugates binding ConA, may be N-glycans and the* T. crassiceps *metacestode N-glycans described by Jang Lee et al. [29] include biantennary and triantennary LacNAc-terminated structures that could explain our MGL1 binding observed. Alternatively or complementarily, the Fuc alpha1-3 GlcNAc antenna might constitute MGL1 targets, as the motif is related to Lewis X. The fact that MGL2 expression was unaffected by MGL1 deletion, suggest that such targets may be useful to trigger better immunity against this helminth.

Here, we add to the knowledge of the role of CLRs in mediating possible immune activation and resistance* in vivo* to the helminth* T. crassiceps*, which may mediate cellular activation via tyrosine phosphorylation. These data agree with those recently reported by Mishra et al. [37], who described a potential role for the MR in inducing proinflammatory responses to the cestode* M. corti*. Thus, it is possible that some CLRs are involved in proinflammatory responses, whereas other CLRs may induce regulatory responses, as has been observed* in vitro* for different helminth antigen-derived molecules that are rich in carbohydrate residues [40, 41]. In fact, several helminth-derived glycoconjugates have been shown to downregulate the inflammatory response of DCs in response to different TLR ligands [41, 42], and a possible signaling through the c-RAF-dependent pathway has recently been proposed as a modulator of TLR-mediated inflammatory responses [40]. All these data support the idea previously suggested by Teale's group [11], indicating that given the complexity of helminth-derived glycomolecules it may be expected that they play distinct roles, including roles in strong regulatory mechanisms as well as the induction of protective responses, which in turn are likely dependent on the specific CLRs that bind such glycoconjugates.

In summary, in this study, we have demonstrated for the first time that cestode-derived molecules are recognized by MGL1 on M*ϕ*s and that such interaction triggers phosphotyrosine-mediated intracellular signaling, which most likely induces inflammatory responses in M*ϕ*s and affects the immune response as well as possible resistance to* T. crassiceps* infection.

## Figures and Tables

**Table 1:** The following primer pairs were used in this study.

Gen	Sequence	Melting temperature	Number of cycles
GAPDH	Forward -CTCATGACCACAGTCCATGC- Reverse -CACATTGGGGGTAGGAACAC-	54°C	35

Arg-1	Forward -CAGAAGAATGGAAGAGTCAG- Reverse -CAGATATGCAGGGAGTCACC-	54°C	35

Ym-1	Forward -TCACAGGTCTGGCAATTCTTCTG- Reverse -TTTGTCCTTAGGAGGGCTTCCTC-	56°C	35

Fizz-1	Forward -GGTCCCAGTGCATATGGATGAGACCATAG- Reverse -CACCTCTTCACTCGAGGGACAGTTGGCAGC-	65°C	35

iNOS	Forward -GCCACCAACAATGGCAACAT- Reverse -AAGACCAGAGGCAGCACATC-	60°C	30
